# Integrating pre-exposure prophylaxis of HIV infection into family planning services: a scoping review

**DOI:** 10.1136/bmjsrh-2021-201356

**Published:** 2022-12-29

**Authors:** Caroline I Gotsche, Petrus S Steyn, Manjulaa Narasimhan, Michelle Rodolph, Rachel Baggaley, James N Kiarie

**Affiliations:** 1 Department of Sexual and Reproductive Health and Research, UNDP/UNFPA/UNICEF/WHO/World Bank Special Programme of Research, Development and Research Training in Human Reproduction (HRP), World Health Organization, Geneve, Switzerland; 2 Faculty of Public Health and Policy, London School of Hygiene & Tropical Medicine, London, UK; 3 Global HIV, Hepatitis and STIs Programmes, World Health Organization, Geneve, Switzerland

**Keywords:** HIV, family planning services, Sexual Health, contraception behavior

## Abstract

**Introduction:**

The aim of this review was to map evidence of integrating pre-exposure prophylaxis (PrEP) for HIV prevention into family planning services. A comprehensive package, using a combination of PrEP and contraceptive delivery, could reduce the number of new HIV infections and/or unintended pregnancies for at-risk women and adolescent girls.

**Methods:**

A scoping literature search was conducted between August 2020 and October 2020. After developing the review question, electronic databases (MEDLINE, Embase, Cochrane Library, Global Health, Web of Science) were systematically reviewed. All types of articles published from 2012 to August 2020 in English were included. The intended outcome was to identify barriers and enablers of integrating services at the client-level and provider-level.

**Results:**

38 articles met inclusion criteria, with 16 from low-and middle-income countries and 22 from high-income countries. Barriers at the client-level included a lack of risk perception associated with low uptake and continuation of PrEP and pill burden; and at the provider-level, barriers included a lack of studies on cost-effectiveness of integrating services and provider training and knowledge. Facilitators included the initiation of PrEP and contraception at the same time and by the same provider or HIV self-testing.

**Conclusion:**

Mapping and synthesising current evidence, this review identified key barriers and facilitators for the integration of PrEP into family planning services for women and adolescent girls. In order to address these factors, more implementation research in a variety of settings is needed to meet women’s sexual and reproductive health needs globally.

**WHAT IS ALREADY KNOWN ON THIS TOPIC**

- Pre-exposureprophylaxis (PrEP) inwomen is an effective HIV prevention option when taken regularly. Providing PrEP in family planning services could contribute to PrEP uptake globally. Understanding how PrEP could be delivered in these services has to be further investigated.

**WHAT THIS STUDY ADDS**

- Multi-level barriers were identified at client level (eg, low risk perception), and at provider-level (eg, lack of provider training and knowledge). Facilitating factors such as HIV self-testing were identified which should be implemented to improve the integration of both services.

**HOW THIS STUDY MIGHT AFFECTRESEARCH, PRACTICE OR POLICY**

- More implementation of research studies are needed from diverse settings on how best to integrate PrEP into family planning services, with current evidence mainly from pilot studies in Kenya and the USA.

## Introduction

Integrating pre-exposure prophylaxis (PrEP) for HIV prevention within family planning (FP) services is an area of emerging interest to researchers and policymakers. Where women and adolescent girls are at increased risk for HIV infection, offering HIV prevention services during visits to FP services provides an important opportunity to reduce both the risk of HIV infection and unplanned pregnancy during clinic visits.^1^ Findings from the Evidence for Contraceptive Options in HIV Outcomes (ECHO) trial showed no significant difference in HIV risk among the contraceptive methods that were evaluated. The aim of this study was to assess whether the risk of acquiring HIV differs between the three different contraceptive methods offered: intramuscular depot medroxyprogesterone acetate, copper intrauterine device, and levonorgestrel implant.^2^ Oral PrEP is an additional effective HIV prevention option for women and adolescent girls.^3^ As a user-controlled HIV prevention method, PrEP could reduce infections occurring disproportionally among women and adolescent girls in southern and East Africa by taking one daily tablet.^4^

Integration is defined as combining different types of services to maximise outcomes and can be bi-directional, with sexual and reproductive health (SRH) services integrated into HIV services and HIV services integrated into SRH services.^5^ Integration of HIV prevention interventions within existing SRH services can improve both access and effectiveness of FP care.^6^ Interest in comprehensive approaches to the integration of SRH, HIV and sexually transmitted infections (STIs) services re-emerged when results of the ECHO trial showed a high incidence of HIV infections and other STIs among women seeking FP services.^2^ Although PrEP was not offered until later in the ECHO trial, it was shown that PrEP use decreased HIV incidence among the women who used it.^7 8^ Since PrEP is not commonly available within FP services, UNAIDS, WHO and the Human Reproduction Programme have jointly called for a rapid introduction of PrEP in FP settings, in locations with high HIV incidence as well as in sites that serve women from key populations.^9^ The objective of this scoping review was to map and synthesise the literature on how PrEP can be integrated into FP services, focusing on potential barriers and enablers. Insights from this review may assist international and national stakeholders to improve the future scale-up of integration of both services.

## Methods

This review was informed by Arksey and O'Malley’s (2005)^10^ methodological framework for scoping reviews. Three authors (CG, PS, JK) developed the research question, search strategy and inclusion criteria. Second checking was performed by two authors (CG, PS), and questionable eligibility of studies was discussed by three authors (CG, PS, JK). The review question was phrased as ‘What evidence exists on the integration of HIV pre-exposure prophylaxis (PrEP) into FP services for women and adolescent girls globally?’. According to the question’s key concepts and synonyms, search strings (online supplemental table 1) were developed that accounted for differences in databases’ search options. Studies included: English language, all article types, all settings, including women and adolescent girls, relating to FP services or care and all populations including key populations (online supplemental table 2).

10.1136/bmjsrh-2021-201356.supp1 Supplementary data

### Search strategy

Between August 2020 and September 2020, five peer reviewed electronic databases were systematically reviewed comprising MEDLINE, Embase, Cochrane Library, Global Health, and Web of Science. To include grey literature the search was also run in Google Scholar. Potential ongoing studies were searched for in PROSPERO (International prospective register of systematic reviews) as well as the Australian New Zealand Clinical Trial Registry (ANZCTR) (online supplemental table 3) The timeframe included all studies from 2012 to August 2020. The rationale for setting the start date in 2012 was the publication date of the Partners PrEP study, which for the first time, showed efficacy of PrEP in women.^3^ Endnote, version X9 (Clarivate, Philadelphia, PA) was used as reference manager. To include articles published after the initial search, email updates were set and reviewed. Further studies were identified through snowballing of experts, references and organisational or institutional websites. In total, 4326 articles were retrieved from databases, and 2651 articles remained after removing duplicates. The following inclusion criteria were applied when screening the material: English language, all article types, all countries, including women and adolescent girls (10–19 years of age, rational: according to WHO definition of adolescence), relating to FP services or care, key populations (eg, sex worker, injecting drug users, trans women). This study focused only on the integration of PrEP into FP services and not into HIV services. By scanning titles and abstracts to identify relevant articles, 2605 articles were removed. Forty-seven full-text articles were assessed for eligibility and nine of those were removed (online supplemental figure 1 11). All types of articles (eg, peer reviewed articles, abstracts and conference posters) were included into the review. Included studies were screened in order to identify patterns and themes arising from the dataset.^12 13^

## Results

### Description of included studies

**Figure 1:**
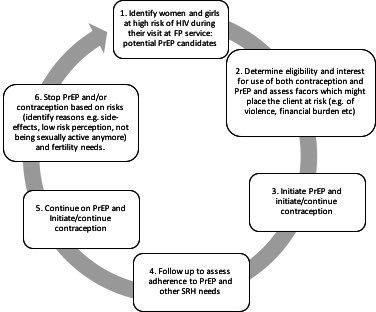
A proposed PrEP and SRH cascade, based on the PrEP cascade supported by WHO. FP, family planning; PrEP, pre-exposure prophylaxis; SRH, sexual and reproductive health.

The search and selection found that 38 articles met the inclusion criteria and were included in the review; 33 of the articles provided primary data (the others were commentaries or opinion pieces without original data). Sixteen articles referred to low- and middle-income countries (LMICs) (13/16 in Kenya) and 22 in high-income countries (HICs) (22/22 in the USA). Studies from LMICs on the integration of PrEP into FP services were primarily published since 2018, studies from the USA primarily since 2016. Online supplemental table 4 provides an overview of all included studies. All included articles referred to oral PrEP only. Of the 16 articles that provided data from LMIC, 11 were from the three implementation studies listed in box 1. In the USA, studies were conducted in various settings. Mainly the first two steps in the PrEP and SRH cascade (figure 1) were investigated in the USA, whereas in LMICs the focus primarily was on steps 3–5 of the cascade.

Box 1

- *PrEP Implementation in Young Women and Adolescents* (PrIYA) programme (n=6) is an implementation programme in Kisumu, Kenya which delivered PrEP to young women at substantial risk of HIV.
- *Zimbabwe National Family Planning Council* (ZNFPC) (n=2) coordinates, takes leadership and supports implementation of integrated FP and related SRH services in Zimbabwe.
- *Prevention Options for Women Evaluation Research* (POWER) study (n=3) intends to understand PrEP use and uptake among women attending FP clinics in Kenya and youth friendly clinics in South Africa.

This review identified multiple inhibiting and facilitating factors when PrEP services were accessed through FP services. Table 1 provides an overview of these barriers and facilitators categorised by PrEP indicators such as interest, uptake and (dis)continuation. Table 2 lists barriers and enablers of integrating PrEP into FP services affecting providers, such as a lack of costing studies and a lack of provider training.

**Table 1:** Barriers and facilitators of PrEP services when PrEP was accessed through FP services

Client-level		
PrEP indicator	Barriers	Facilitators
Awareness	Kenya: AGYW ≤18 years of age were less aware of PrEP	Kenya: AGYW with regular employment; AGYW engaged in transactional sex or condomless sex in the last 6 months were more likely to take up PrEP
Uptake	Kenya: Low perceived risk for HIV reduced PrEP uptake Zimbabwe: Fear of pill burden, wanting partner’s consent or fearing partner’s reaction to PrEP, satisfied with current method of HIV prevention USA: PrEP stigma reduced uptake USA: Perception of PrEP being too new and mistrust in the medical community reduced uptake	Kenya: Unmarried women, women with an HIV positive partner, women >24 years of age were more likely to take up PrEP Zimbabwe: High HIV risk perception, preference for PrEP over other HIV prevention methods, perceived severity of living with HIV, confidence in PrEP USA: Clinical tools for provider and education material for clients increased PrEP uptake
Continuation	Zimbabwe: Unaccustomed to taking pills, religious issues, travel out of town, clinic schedule/hours, lack of transport funds, misunderstanding dosing guidance, side effects Kenya: Low perceived risk of acquiring HIV, finding out partner was HIV negative, side effects, pill burden, fear of intimate partner violence reduced continuation of PrEP	Zimbabwe: Focus on original motivation, establishing daily pill routine, accessible PrEP pill storage, planning ahead before travel out of town, partner or facility support impacted positively on PrEP continuation

AGYW, adolescent girls and young women; FP, family planning; PrEP, pre-exposure prophylaxis.

**Table 2:** Barriers and facilitators affecting providers when PrEP was accessed through FP services

Provider-level		
	Barriers	Facilitators
Costs	Lack of costing studies	Kenya: Reducing costs through (1) postponing creatinine testing and (2) prioritising PrEP delivery to clients at high HIV risk
Workforce	Kenya: Increased workload of healthcare worker, physical space constraints, drug stockouts, inaccurate PrEP knowledge	Kenya: Task sharing, facility-specific client flow modifications (eg, fast tracking), myths busting groups
Provider training	Kenya: Lack of provider training USA: Lack of provider training	USA: 1.5 hour PrEP information training improved providers’ PrEP knowledge

FP, family planning; PrEP, pre-exposure prophylaxis.

Furthermore, when available, characteristics of implementation strategies were specified in the results section.

Themes that arose from the dataset included client-level and provider-level barriers and facilitators of delivering PrEP in settings where FP services were provided.

### Client-level barriers and facilitators affecting PrEP services when integrated into FP services

#### Awareness and knowledge of PrEP

A survey conducted by Sila *et al* found that awareness of PrEP (89%) was high among high-risk adolescent girls and young women (AGYW) (n=470) attending four FP services in Kisumu, Kenya.^14^ Although overall awareness of PrEP among clients was high, it was lower among AGYW ≤18 years of age compared with those >18 years, which can be perceived as a barrier to awareness. Enablers of awareness included regular employment, engagement in transactional sex in the last 6 months, and engagement in condomless sex in the last 6 months.^14^ In the USA, figures on awareness among women (general population and at high risk) attending FP services varied considerably across studies.^15–17^ O’Connell and Critini explored the feasibility of integrating routine PrEP counselling in a high-volume FP clinic with no previous PrEP experience using a Women’s PrEP Counselling Checklist (WPCC) tool developed to structure and standardise each counselling session. Clients who received WPCC-guided counselling had significantly higher knowledge scores and more enhanced PrEP acceptability than their peers receiving unguided counselling.^18^

#### Uptake of PrEP

In a qualitative study, Gombe *et al* 19 studied key barriers (fear of pill burden such as size of pill or difficulties with adherence, wanting partner’s consent or fearing of partner reaction, or feeling satisfied with current method of HIV prevention) and enablers (high HIV risk perception, preference for PrEP over other HIV prevention measures, perceived severity of living with HIV, and confidence in PrEP) associated with uptake of PrEP in two semi-public Zimbabwe National Family Planning Council (ZNFPC) clinics.^19^ In the USA, Sales *et al* found that delivering PrEP through FP services, which are already trusted by black adolescent and young adult women, can have a positive impact on drug adherence and uptake and therefore act as an enabler of uptake.^20^ In their survey, Calabrese *et al* 15 identified stigma as an important structural barrier reducing PrEP uptake.^15^ Using the RE-AIM (Reach, Efficacy, Adoption, Implementation, Maintenance) evaluation framework, Brant *et al* 21 showed a low PrEP uptake (6%) when integrated into FP care. Enablers, such as clinical tools for providers and client education materials, were provided and increased uptake. The RE-AIM framework is a widely used tool to assess the quality, feasibility, and public health impact of a health intervention.^21^ Overall, figures of PrEP uptake varied across studies. In their qualitative study, Gombe *et al* piloted PrEP integration in an urban FP clinic in Zimbabwe in 2018, and found an uptake of 4.1% that was influenced by partners’ or families’ support, by healthcare workers’ knowledge and attitudes, as well as participants’ awareness of PrEP.^22^ This aligns with the figures of Sila *et al* 14 who also found a low uptake of 4% despite the high awareness of PrEP (89%). In their study, 24% of AGYW who declined PrEP had a high HIV risk score^14^ ≥5 (VOICE score, Vaginal and Oral Interventions to Control the Epidemic (VOICE) study).^23^ Thus, low risk perception is considered to be an important barrier to PrEP uptake. In Kenya, Mugwanya *et al* integrated routine HIV testing and counselling for PrEP into FP clinics which was delivered by a PrEP dedicated-nurse. Results showed that PrEP uptake was 22% among HIV-negative women, overall, and 16% among AGYW (n=1271). Facilitators for an uptake of PrEP among study participants included unmarried women, women who had an HIV positive partner, and women >24 years old.^24^ As indicated in Sila *et al*’s study, a woman’s low perceived risk for HIV was an important reason for low PrEP uptake.^14^ Deviating from other study’s figures, early results from the Prevention Options for Women Evaluation Research (POWER) cohort study indicated a rather high uptake, with 90% of enrolled AGYW (n=540) agreeing to initiate PrEP. In this abstract, however, no further information on potential reasons for a higher uptake was provided.^25^

#### Continuation of PrEP

In Zimbabwe, Gombe *et al* 19 investigated factors affecting continuation of PrEP for women accessing PrEP through FP clinics. Common barriers included being unaccustomed to taking pills, religious issues, travel out of town, clinic schedule/hours, lack of transport funds, misunderstanding dosing guidance or side-effects. Facilitators included focusing on original motivation, establishing a daily pill routine, accessible PrEP storage, planning ahead before travel out of town, and the promotion of partner or facility support.^19^ In Jilinde, a 4-year PrEP scale-up project in Kenya, FP and PrEP services and counselling were offered during the same session by the same provider, and follow-up appointments were synchronised. AGYW initiating PrEP and FP at the same time (39.4%) had higher month continuation rates after 1 month, compared with AGYW who only initiated PrEP (36.2%).^26^ Data on continuation and discontinuation remained scarce with more evidence coming from LMIC than from HIC. Early results from the POWER study showed that 50% of those starting PrEP discontinued within 1 month and another 25% by 3 months.^25^ Mugwanya *et al*’s findings revealed that PrEP discontinuation was high in women who accessed PrEP in FP clinics. After 1, 3, and 6 months post-initiation, only 41%, 24%, and 15% of all women, respectively, continued using PrEP. Most frequent reasons for discontinuation were a low perceived risk of acquiring HIV (25%), finding out that their male partner was HIV negative (24%), side effects (20%), and pill burden (17%); additionally, 7% of women reported discontinuing PrEP due to fear of intimate partner violence (IPV).^27^

### Provider-level barriers and facilitators affecting PrEP services

#### Costs

Roberts *et al* 28 measured incremental costs of integrating PrEP into FP services and explored cost implications of service delivery modifications in the context of the PrEP Implementation in Young Women and Adolescents (PrIYA) programme.^28^ Before PrEP is recommended, testing for HIV and other STIs as well as a renal function test is required.^29^ Several measures were identified to reduce costs and burden on providers and may therefore act as facilitators of integrating services: (1) postponing the renal function or creatinine testing to the first follow-up visit; and (2) targeting people at higher risk. These measures reduced costs by 8% and 14%, respectively (US$26.52 cost/client/month). Additionally, costs decreased to US$16.54/client/month when implementation was delivered by the ministry of health personnel.^28^

#### Workforce

Beima-Sofie *et al* 30 found that healthcare workers (HCW) in Kenya felt overburdened by the increased workload when PrEP was integrated into FP services. Barriers reported by HCW included physical space constraints, drug stockouts, inaccurate PrEP knowledge, and lack of PrEP delivery training. HCW strategies to optimise delivery included task sharing (nurses to HIV testing service providers), facility-specific client flow modifications such as fast-tracking PrEP clients to reduce waiting times, and myth-busting during group health talks to reduce individual time spent counselling clients.^30^ In the USA, Seidman *et al* conducted a national survey which identified a lack of provider training as one of the main structural barriers to implementing PrEP, with <50% of FP providers answering questions on PrEP correctly.^31^ Sales *et al* showed significant improvement of providers’ PrEP knowledge and confidence to identify eligible women after providing a 1.5 hour PrEP information training in public FP clinics in a high HIV prevalence area in the USA.^32^

## Discussion

This scoping review aimed to map and synthesise the existing literature on the integration of PrEP into FP services for women and adolescent girls; special emphasis was dedicated to barriers and facilitators when PrEP was accessed through FP services. Findings revealed multi-level barriers affecting the PrEP and SRH cascade (Figure 1) and its indicators at the client- as well as the provider-level. Identified barriers and enablers may affect the integration of PrEP into FP services and therefore have to be addressed by future implementation research studies and policies.

### Client-level barriers and facilitators

#### Awareness and knowledge of PrEP

In a Kenyan setting, awareness was significantly lower in younger women (<18 years), highlighting the need to find ways of specifically supporting this age group.^33^ The ECHO trial showed that HIV incidence was higher in women <25 years old, which highlights the urgency of HIV prevention efforts in that age group and the impact PrEP use could have in AGYW.^2^

#### Uptake and continuation of PrEP

Results identified a reduced risk perception to be a major barrier of PrEP interest,^34^ as well as uptake and continuation.^14 27 35^ This corresponds with Ngure *et al*’s findings revealing that a high risk perception was found to be a motivator for PrEP initiation and continuation.^36^ Garfinkel *et al* 37 found that women’s HIV risk perception was shaped by both behavioural and structural risk factors, including IPV, sexual coercion, having multiple sexual partners, and trading sex.^37^ Therefore, targeting women who face IPV or who trade sex may be important to address the lack of risk perception among particularly vulnerable populations. Women’s perception of their own risk is a critical component of PrEP uptake^38^ and this review’s findings showed that women’s perceived and actual risk were often misaligned,^14 39 40^ requiring refined approaches to ensure a woman’s perceived risk of HIV acquisition meets her actual risk. In a matched-cluster randomised controlled trial, Thomas *et al* are currently investigating whether an interactive tablet-based education intervention can correct risk perception.^41^ Approaches as to how to achieve an alignment of women’s risk perception and their actual risk are not yet well understood and more research is needed.

As a key barrier, this review revealed that particularly younger women were less likely to initiate or continue using PrEP. Mugwanya *et al* showed that being younger than 24 years of age decreased the likelihood of PrEP uptake and continuation.^19^

Across settings, results identified pill burden and not being accustomed to taking pills as one of the major barriers for uptake or continuation of PrEP at the client-level.^14 19 24 34 42^ This aligned with recent findings from Kenya revealing that AGYW found it challenging to keep up with daily PrEP pill-taking.^43^ Challenges to adhere to daily medication are not unique to PrEP.^44^ However, PrEP can only prevent HIV in women when taken consistently as prescribed.^45^ Therefore, overcoming this barrier is essential to increase uptake and continuation of oral PrEP. According to Gombe *et al*, establishing a daily pill routine, as well as accessible PrEP pill storage, were facilitating factors.^19^

Follow-up visits, to test for HIV regularly and to provide support to continue PrEP, were also considered a barrier to PrEP uptake, which may also have an impact on the integration of PrEP in FP services.^34 42^ Tools such as HIV self-testing (HIVST) may facilitate the integration of PrEP into FP services on a client-level by reducing the number of clinic visits, but also on a provider-level by saving health workers time.^46 47^ A study in Kenya found that using HIVST in PrEP delivery among FP clients was feasible and had the potential to simplify its delivery at the client-level.^47^ This aligns with current recommendations by WHO to integrate HIVST into contraceptive clinics as it is highly acceptable, feasible and empowering for women.^9^

### Provider-level barriers and facilitators

This review identified a lack of costing studies on the integration of PrEP into FP services. Roberts *et al*’s costing study was the only study that addressed this. It found that integrating PrEP into other medical or non-medical services may be an efficient strategy to reach priority populations and may have low incremental costs. However, the authors’ highlighted results were context-specific and not easily transferable to other settings.^28^

The facilitating role that trained providers play in improving the integration of PrEP into FP settings was emphasised by different studies across settings^30–32 37^ and is aligned with WHO recommendations on how to prevent HIV in women using FP services.^9^ Existing literature highlights that provider training is essential in order to integrate services and is often challenging to achieve.^48 49^ This review identified provider training and knowledge of PrEP to be an important barrier^30 31^ and at the same time facilitator^18 32^ when PrEP services are integrated into FP services. Facilitators

included task sharing or facility-specific client flow modifications (eg, fast tracking). This is supported by Bhavaraju *et al* claiming that changes in policies on task sharing may be needed to enable the integration of PrEP into FP services.^50^

Inhibiting and enabling factors identified by this review impact on the integration of services and ultimately limit the impact oral PrEP may have when provided within FP settings. Exploiting potential synergies of both services may help to overcome barriers and enablers presented above.

#### Synergy of services

Synergy is defined as ‘the combined power of a group of things when they are working together that is greater than the total power achieved by each working separately’.^51^ Synergies may arise when PrEP and contraceptive methods are offered simultaneously. To appreciate the role synergies may play in the integration of PrEP into FP services we adapted the PrEP cascade supported by WHO, and integrated the SRH aspects.^38^ In the context of integrating PrEP into FP services, there are benefits of these synergies.^21 26^ For example, both contraceptive methods and PrEP have to consider eligibility, client choice, contraindications and time period needed to use PrEP.^52^ This review included a study from Were *et al* 26 that suggested delivering PrEP combined with FP services to AGYW might work synergistically to improve continuation for both interventions.^26^ They found that when PrEP and FP were initiated concurrently at the same visit and by the same provider, discontinuation after months 1 and 3 was less likely. However, results of this review indicated that characteristics of these synergies and how they may impact positively on PrEP and FP outcome measures has not yet been researched in depth.

#### Recommendations for future research

This review found that research was primarily limited to Kenya and the USA, requiring more implementation of research from a variety of settings to ensure generalisability.

Indicators alongside the PrEP and SRH cascade help to assess and monitor a programme’s effectiveness and to identify bottlenecks and opportunities for improvement.^38 52^ Future implementation of research studies that cover all steps of the cascade are needed in order to inform the intervention’s scale-up at large. For example, among included studies, data on continuation of PrEP when it was provided in the context of FP services was limited in both LMIC and HIC. When investigated, it was low, further declining from 1 to 3 months post-initiation.^24^ Future studies on this should seek to understand why women discontinue PrEP, and to support and advise woman who may want to stop and re-start PrEP. It is also important to understand reasons for stopping PrEP—for example, side-effects such as the ‘start-up syndrome’ sometimes experienced during the first month of PrEP use, no longer feeling at risk for HIV acquisition, or other reasons. Tailoring an adequate response that addresses these reasons will help ensure a PrEP programme’s effectiveness.^38^

In the context of successful integration of services, the improvement of a programme’s cost-effectiveness through integration is often assumed.^53^ However, whether this applies to the integration of PrEP into FP services is not yet well understood—calling for future research with this particular focus.

More information is needed to which extent potential facilitators may impact positively on the integration of PrEP into FP services. For example, HIVST is recommended in FP settings. In the context of PrEP delivery within these contexts, however, data especially on cost-effectiveness and client acceptability and feasibility are needed.

#### Limitations

The amount of novel data included into this review is limited since all types of articles (eg, commentaries) were included. Conference poster and abstracts were also included in this review, which made a systematic quality assessment of all studies unfeasible. Only material in English was reviewed. In LMIC, primary data mainly stemmed from three studies (PrIYA, ZNFPC and POWER) which may have reduced the significance for this review’s results. Most studies were based in two countries, Kenya and the USA, which reduced generalisability of results and points to the need for research in other countries.

Most of the included studies examined PrEP programmes delivered in the context of FP services by measuring PrEP indicators. This review found that figures on PrEP indicators (eg, uptake or continuation) varied considerably across settings, and reasons for the variation remained unclear. For example, in LMIC, figures on uptake varied widely, ranging from 4%^14^ to 90%^25^ of study participants who initiated PrEP at their current FP visit. As Dunbar *et al* 52 discussed in their review, interpretation of PrEP indicator measures (eg, uptake) may be impeded due to an interchangeable use of various indicators with different meanings and a lack of a common definition. Although possible, the variation of indicators across studies may be, at least in part, due to inconsistency in terminology, but it could not be ultimately answered by the given data. Promoting guidance on standardised measures and definitions across the PrEP and SRH cascade will be important to allow the comparison of indicators across settings.^52^

## Conclusion

As a mapping and synthesis of currently available evidence in this field, this review identified barriers and facilitators for the integration of PrEP into FP services for women and adolescent girls. Multiple barriers along the PrEP and SRH cascade have an impact on clients (low-risk perception, young age) and providers (lack of training and knowledge) and indicate other areas needing further research. There is a need to enhance the integration of PrEP into FP services, including self-care interventions such as HIV self-testing or enhancing training of providers. Data on the integration of PrEP into FP services was limited, with most studies from Kenya and the USA. To prepare global scale-up of PrEP in FP services successfully, future studies have to be conducted in a variety of settings. As part of a comprehensive package, using a combination of PrEP and contraceptive delivery could reduce the number of new HIV infections and/or unintended pregnancies for at-risk women and girls.
