# Supplementary material for: Integrating pre-exposure prophylaxis of HIV infection into family planning services: a scoping review
Source: BMJ Sex Reprod Health. 2022 Dec 29;49(3):210–8. doi: 10.1136/bmjsrh-2021-201356 (PMC10359582; doi:10.1136/bmjsrh-2021-201356)

SUPPLEMENT - Integrating PrEP into Family Planning Services - A scoping review

Supplementary table 1: Details Search Strategy

Name of data base	Search strategy	Number of records retrieved
**MEDLINE**	1 (PrEP or preexposure prophylaxi* or pre-exposure prophylaxi*).mp. (4311)<br>2 Pre-Exposure Prophylaxis/ (1961)<br>3 1 or 2 (4311)<br>4 (family planning or contracept* or birth spac*).mp. (15827)<br>5 Family Planning Services/ (1741)<br>6 contraception/ or hormonal contraception/ or long-acting reversible contraception/ (2489)<br>7 ((sexual and reproductive) adj3 (health or healthcare)).mp. (2889)<br>8 Reproductive Health Services/ (602)<br>9 reproductive health/ or sexual health/ (2498)<br>10 exp HIV/ (16290)<br>11 exp HIV Infections/ (42748)<br>12 (hiv or hiv-1 or hiv-2 or human immunodeficiency virus*).mp. (91639)<br>13 or/4-12 (110132)<br>14 (wom?n or girl* or AGYW or adolescent* or youth).mp. (689470)<br>15 exp Women/ (4984)<br>16 Adolescent/ (307962)<br>17 14 or 15 or 16 (689470)<br>18 3 and 13 and 17 (1094)<br>19 limit 18 to (English language and yr="2012 -Current") (1081)	1081
**EMBASE**	1 (PrEP or preexposure prophylaxi* or pre-exposure prophylaxi*).mp. (12854)<br>2 Pre-Exposure Prophylaxis/ (4392)<br>3 1 or 2 (12854)<br>4 (family planning or contracept* or birth spac*).mp. (165672)<br>5 family planning/ (40027)<br>6 contraception/ or hormonal contraception/ or long-acting reversible contraception/ (55412)<br>7 ((sexual and reproductive) adj3 (health or healthcare)).mp. (10082)<br>8 exp reproductive health/ (17489)<br>9 exp Human immunodeficiency virus/ (196001)<br>10 exp Human immunodeficiency virus infection/ (377192)<br>11 (hiv or hiv-1 or hiv-2 or human immunodeficiency virus*).mp. (502456)<br>12 or/4-11 (721124)	556

	13 (wom#n or girl* or AGYW or adolescent* or youth).mp. (3453070)<br>14 female/ (9737405)<br>15 adolescent/ (1663970)<br>16 13 or 14 or 15 (10506992)<br>17 3 and 12 and 15 (570)<br>18 limit 17 to (English language and yr="2012 -Current") (556)	
**Web of Science**	# 1 (TS=(PrEP OR "preexposure prophylaxi*" OR "pre-exposure prophylaxi*")) AND LANGUAGE: (English) Indexes=SCI-EXPANDED, SSCI, A&HCI, CPCI-S, CPCI-SSH, ESCI Timespan=2012-2020 (6944)<br># 2 (TS=(family planning OR contracept* OR birth spac*))<br>AND LANGUAGE: (English) Indexes=SCI-EXPANDED, SSCI, A&HCI, CPCI-S, CPCI-SSH, ESCI Timespan=2012-2020 (42381)<br># 3 (TS=Family Planning Services) AND LANGUAGE: (English) Indexes=SCI-EXPANDED, SSCI, A&HCI, CPCI-S, CPCI-SSH, ESCI Timespan=2012-2020 (5752)<br># 4 (TS=reproductive healthcare) AND LANGUAGE: (English) Indexes=SCI-EXPANDED, SSCI, A&HCI, CPCI-S, CPCI-SSH, ESCI Timespan=2012-2020 (2813)<br># 5 (TS=(HIV OR HIV infect*)) AND LANGUAGE: (English) Indexes=SCI-EXPANDED, SSCI, A&HCI, CPCI-S, CPCI-SSH, ESCI Timespan=2012-2020 (148668)<br># 6 (TS=(wom?n or girl* or AGYW or adolescent* or youth)) AND LANGUAGE: (English) Indexes=SCI-EXPANDED, SSCI, A&HCI, CPCI-S, CPCI-SSH, ESCI Timespan=2012-2020 (914740)<br># 7 (#5 OR #4 OR #3 OR #2) AND LANGUAGE: (English) Indexes=SCI-EXPANDED, SSCI, A&HCI, CPCI-S, CPCI-SSH, ESCI Timespan=2012-2020 (190857)<br># 8 (#7 AND #6 AND #1) AND LANGUAGE: (English) Indexes=SCI-EXPANDED, SSCI, A&HCI, CPCI-S, CPCI-SSH, ESCI Timespan=2012-2020 (1696)	1696
**Global Health**	1 (PrEP or preexposure prophylaxi* or pre-exposure prophylaxi*).mp. (2503)<br>2 (family planning or contracept* or birth space*).mp. (19486)<br>3 exp family planning/ (10370)<br>4 contraceptives/ or contraception/ or injectable contraceptives/ or oral contraceptives/ (10561)<br>5 ((sexual and reproductive) adj3 (health or healthcare)).mp. (3950)<br>6 exp reproductive health/ (8451)<br>7 exp sexual health/ (2938)<br>8 exp human immunodeficiency viruses/ (179862)<br>9 exp HIV infections/ (147094)<br>10 (hiv or hiv-1 or hiv-2 or human immunodeficiency virus*).mp. (186902)<br>11 or/2-10 (216594)<br>12 (wom#n or girl* or AGYW or adolescent* or youth).mp. (454945)<br>13 exp women/ (255165)<br>14 exp adolescents/ (64805)<br>15 12 or 13 or 14 (455041)<br>16 1 and 11 and 15 (652)	612

	17 limit 16 to (English language and yr="2012 -Current") (612)	
**Cochrane Library**	#1 (PrEP or "preexposure prophylaxi*" or "pre-exposure prophylaxi*"):ti,ab,kw (Word variations have been searched) (1752)<br>#2 MeSH descriptor: [Pre-Exposure Prophylaxis] explode all trees (163)<br>#3 #1 or #2 (1752)<br>#4 ("family planning" or contracept* or birth spac*):ti,ab,kw (13440)<br>#5 MeSH descriptor: [Family Planning Services] explode all trees (215)<br>#6 MeSH descriptor: [Contraception] explode all trees (448)<br>#7 MeSH descriptor: [Reproductive Health Services] explode all trees (449)<br>#8 MeSH descriptor: [Sexual Health] explode all trees (38)<br>#9 MeSH descriptor: [HIV] explode all trees (3017)<br>#10 MeSH descriptor: [HIV Infections] explode all trees (12355)<br>#11 (hiv or hiv-1 or hiv-2 or human immunodeficiency virus*):ti,ab,kw (26578)<br>#12 #4 or #5 or #6 or #7 or #8 or #9 or #10 or #11 (39893)<br>#13 (wom?n or girl* or AGYW or adolescent* or youth):ti,ab,kw (273179)<br>#14 MeSH descriptor: [Women] explode all trees (604)<br>#15 MeSH descriptor: [Adolescent] explode all trees (102500)<br>#16 #13 or #14 or #15 (273179)<br>#17 #3 and #12 and #16 with Cochrane Library publication date from Aug 2012 to Aug 2020 (350)	1
**Google scholar**	(Preexposure prophylaxis OR Pre-exposure prophylaxis OR PrEP) AND (women OR woman OR "adolescent girls and young women" OR AGYW OR girls OR youth)) AND ("family planning" OR contracept* OR birth spac* OR "sexual health" OR "reproductive health" OR HIV OR "human immunodeficiency virus" OR HIV-1 OR HIV-2) Limited to 2012-2020	380
**TOTAL amount of articles retrieved from databases**		4326
**TOTAL amount of articles after removing duplicates**		**2651**

Supplementary table 2 Inclusion Criteria

**Inclusion criteria:**	English language, all article types, all settings, including women and adolescent girls (10-19 years of age, rational: according to WHO definition of adolescence), relating to family planning services or care, key populations (e.g. sex worker, injecting drug users, trans women).
**Exclusion criteria:**	pregnant and postpartum women (rational: pregnant and postpartum women rarely attend family planning services), basic science (e.g. clinical or pharmacological research/ trials on PrEP efficacy, not containing PrEP as main intervention, studies that omit important details (e.g. title or author) or focus on other diseases than HIV.

Supplementary table 3 Ongoing Studies

Ongoing studies

#	Investigator	Title	Country	Study type	Protocol
**A**	Sales et al.	Pre-Exposure Prophylaxis Integration into Family Planning Services at Title X Clinics in the Southeastern United States: A Geographically-Targeted Mixed Methods Study (Phase 1 ATN 155)	US	A Geographically-Targeted Mixed Methods Study	Jmir Research Protocols 8(6): 10.
**B**	Celum et al.	A Cohort for Evaluation of Open-label PrEP Delivery Among Kenyan and South African Women: The POWER Cohort, study ID STUDY00000950	Kenya, South Africa	Cohort study	NCT03490058
**C**	Kohler et al.	Standardized Patient Encounters to Improve PrEP Counselling for Adolescent Girls and Young Women in Kenya (PrIYA-SP)	Kenya	Behavioural: Clinician training intervention	NCT03875950

Supplementary table 4 Included Studies

#	Author & Year	Title	Journal	Country	Article type/ Study type
		**Low-and middle-income countries**			
**1**	Gombe et al. (2020)	Key barriers and enablers associated with uptake and continuation of oral pre-exposure prophylaxis (PrEP) in the public sector in Zimbabwe: Qualitative perspectives of general population clients at high risk for HIV	PLoS One	Zimbabwe	Full article/ Qualitative study
**2**	Sila et al. (2020)	High Awareness, Yet Low Uptake, of Pre-Exposure Prophylaxis Among Adolescent	AIDS Patient Care STDS	Kenya	Full article/ Cross-sectional study conducted as follow-on study

		Girls and Young Women Within Family Planning Clinics in Kenya			
**3**	Wanga et al. (2020)	Uptake and impact of facility-based HIV self-testing on PrEP delivery: a pilot study among young women in Kisumu, Kenya	J Int AIDS Soc	Kenya	Full article/ Implementation research study<br>Cohort study
**4**	Were et al. (2020)	Oral pre-exposure prophylaxis (PrEP) and family planning (FP) integration to improve PrEP continuation among adolescent girls and young women (AGYW) in Kenya	J Int AIDS Soc	Kenya	Conference abstract/ no specifics provided
**5**	Beima-Sofie et al. (2019)	Implementation challenges and strategies in integration of PrEP into maternal and child health and family planning services: Experiences of frontline healthcare workers in Kenya	J Int AIDS Soc	Kenya	Conference presentation/ Qualitative study
**6**	Celum et al. (2019)	HIV pre-exposure prophylaxis for adolescent girls and young women in Africa: from efficacy trials to delivery	J Int AIDS Soc	Kenya, South Africa, Tanzania, Zimbabwe	Commentary Review/ Na
**7**	Hodges-Mameletzis et al. (2019)	Pre-Exposure Prophylaxis for HIV Prevention in Women: Current Status and Future Directions	Drugs	n/a	Narrative review/ Na
**8**	Mugwanya et al. (2019)	Integrating preexposure prophylaxis delivery in routine family planning clinics: A feasibility programmatic evaluation in Kenya	PLoS Medicine	Kenya	Full article/ Pilot open-label, “real-world” implementation study
**9**	Pintye et al. (2019)	Frequent detection of tenofovir-diphosphate among young Kenyan women in a real-world PrEP implementation program	J Int AIDS Soc	Kenya	Brief report/ Pilot open-label, “real-world” implementation study
**10**	Roberts et al. (2019)	The role of costing in the introduction and scale-up of HIV pre-exposure prophylaxis: evidence from integrating PrEP into routine maternal and child health and family planning clinics in western Kenya	J Int AIDS Soc	Kenya	Full article/ Costing study
**11**	Dunbar et al. (2018)	Understanding and measuring uptake and coverage of oral pre-exposure prophylaxis delivery among adolescent girls and young women in sub-Saharan Africa	Sex Health	DREAMS countries (Kenya, Lesotho, Malawi,	Full article/ Literature review

				Mozambique, South Africa, Swaziland, Tanzania, Uganda, Zambia, Zimbabwe)	
**12**	Gombe et al. (2018)	Integrating oral HIV pre-exposure prophylaxis (PrEP) in a public family planning facility and youth centre to inform national roll out in Zimbabwe	J Int AIDS Soc	Zimbabwe	Conference abstract/ Qualitative study
**13**	Mugwanya et al. (2018)	Uptake of PrEP within clinics providing integrated family planning and PrEP services: Results from a large implementation program in Kenya	J Int AIDS Soc	Kenya	Conference abstract/ Pilot open-label, “real-world” implementation study
**14**	Mugwanya et al. (2018)	One-month PrEP Continuation Among Adolescent Girls and Young Women in Routine Maternal Child Health and Family Planning Clinics	AIDS Res Hum Retroviruses	Kenya	Conference abstract/ Pilot open-label, “real-world” implementation study
**15**	Omollo et al. (2018)	PrEP delivery platforms: Are family planning clinics an option?	AIDS Res Hum Retroviruses	Kenya	Conference abstract/ Open-label prospective study
**16**	Rousseau-Jemwa et al. (2018)	A comparative study of risk among adolescent girls and young women who accept or decline PrEP uptake from a community-based mobile clinic	22nd international AIDS conference	Kenya/ South Africa	Conference abstract/ Open label PrEP implementation study
		**High-income countries**			
**17**	Brant et al. (2020)	Integrating HIV Pre-Exposure Prophylaxis into Family Planning Care: A RE-AIM Framework Evaluation	AIDS Patient Care STDS	USA, Washington DC	Full article/ Prospective cohort study
**18**	Calabrese et al. (2020)	Contraception as a Potential Gateway to Pre-Exposure Prophylaxis: US Women's Pre-Exposure Prophylaxis Modality Preferences Align with Their Birth Control Practices	AIDS Patient Care STDS	USA, Connecticut	Full article/ Cross-sectional study

**19**	Fields et al. (2020)	Understanding Opportunities to Discuss HIV Prep in Contraceptive Counselling Services Provided to Adolescent Women	J Adolesc Health	USA	Conference poster/ Secondary analysis study
**20**	Johnson et al. (2020)	Awareness and Intent to Use Pre-exposure Prophylaxis (PrEP) Among African American Women in a Family Planning Clinic	J Racial Ethn Health Disparities	USA	Full article, Cross-sectional study
**21**	O'Connell et al. (2020)	The Impact of HIV Pre-Exposure Prophylaxis (PrEP) Counselling on PrEP Knowledge and Attitudes among Women Seeking Family Planning Care	J Womens Health	USA, Philadelphia	Full article, Cross-sectional study Survey
**22**	O'Malley et al. (2020)	Intimate Partner Violence, HIV Pre-Exposure Prophylaxis (PrEP) Acceptability, and Attitudes About Use: Perspectives of Women Seeking Care at a Family Planning Clinic	AIDS Behav	USA, Pittsburgh	Full article, Mixed-methods study
**23**	Sales et al. (2020)	Where can Southern girls go for prep? Examining the PrEP-providing practices of Title-X funded family planning clinics across the Southern US	J Adolesc Health	USA	Abstract/ Explanatory, sequential mixed-methods study
**24**	Unger et al. (2020)	Providing Pre-exposure Prophylaxis in Family Planning Centers: A Survey of Provider Knowledge and Attitudes	J Assoc Nurses AIDS Care	USA	Full article/ Cross-sectional study
**25**	Carley et al. (2019)	Interest in Pre-exposure Prophylaxis (PrEP) for HIV is Limited Among Women in a General Obstetrics & Gynaecology Setting	AIDS Behav	USA, Louisiana	Full article/ Cross-sectional study
**26**	Sales et al. (2019)	Impact of PrEP Training for Family Planning Providers on HIV Prevention Counselling and Patient Interest in PrEP in Atlanta, Georgia	J Acquir Immune Defic Syndr	USA, Atlanta	Full article/ Implementation study
**27**	Sales et al. (2019)	Patient recommendations for PrEP information dissemination at family planning clinics in Atlanta, Georgia	Contraception	USA, Atlanta	Full article/ Cross-sectional study
**28**	Calabrese et al. (2018)	HIV Pre-Exposure Prophylaxis Stigma as a Multidimensional Barrier to Uptake Among Women Who Attend Planned Parenthood	J Acquir Immune Defic Syndr	USA, Connecticut	Full article/ Cross-sectional study
**29**	Haider et al. (2018)	Examining knowledge of and attitudes toward preexposure prophylaxis (PrEP) among HIV-vulnerable women in a Chicago-based family planning clinic	Contraception	USA, Chicago	Abstract/ Mixed-methods study

**30**	Koren et al. (2018)	HIV Pre-Exposure Prophylaxis and Women: Survey of the Knowledge, Attitudes, and Beliefs in an Urban Obstetrics/Gynaecology Clinic	AIDS Patient Care STDS	USA, Philadelphia	Full article/ Cross-sectional study Survey
**31**	Pollock et al. (2018)	Role of Preexposure Prophylaxis in the Reproductive Health of Women at Risk for Human Immunodeficiency Virus Infection	Obstet Gynecol	USA	Current commentary/ Na
**32**	Scott et al. (2018)	Knowledge and acceptability of HIV preexposure prophylaxis (PrEP) among women presenting for family planning services in a high-prevalence community	Contraception	USA, Washington DC	Abstract/ Cross-sectional study
**33**	Seidman et al. (2018)	Family planning providers' role in offering PrEP to women	Contraception	USA	Review article/ Na
**34**	Garfinkel et al. (2017)	Predictors of HIV-related risk perception and PrEP acceptability among young adult female family planning patients	AIDS Care	USA, Baltimore City	Full article/ Cross-sectional study
**35**	Sanders et al. (2017)	Integration of prep in an academic adolescent clinic & impact of prep use on sexually transmitted infection (STI) rates	Sex Transm Infect	USA, Baltimore City	Abstract/ No specifics provided
**36**	Seidman et al. (2016)	United States family planning providers' knowledge of and attitudes towards preexposure prophylaxis for HIV prevention: a national survey	Contraception	USA	Full article/ Cross-sectional study
**37**	Seidman et al. (2016)	Women's Knowledge of, Interest in, and Eligibility for HIV Pre-exposure Prophylaxis at Family Planning Clinics in Northern California	AIDS Res Hum Retroviruses	USA, Northern California	Meeting abstract/ Cross-sectional study
**38**	Seidman et al. (2016)	Integrating Preexposure Prophylaxis for Human Immunodeficiency Virus Prevention Into Women's Health Care in the United States	Obstet Gynecol	USA,	Commentary/ Na

Na/ not applicable

Figure a: PRISMA flowchart

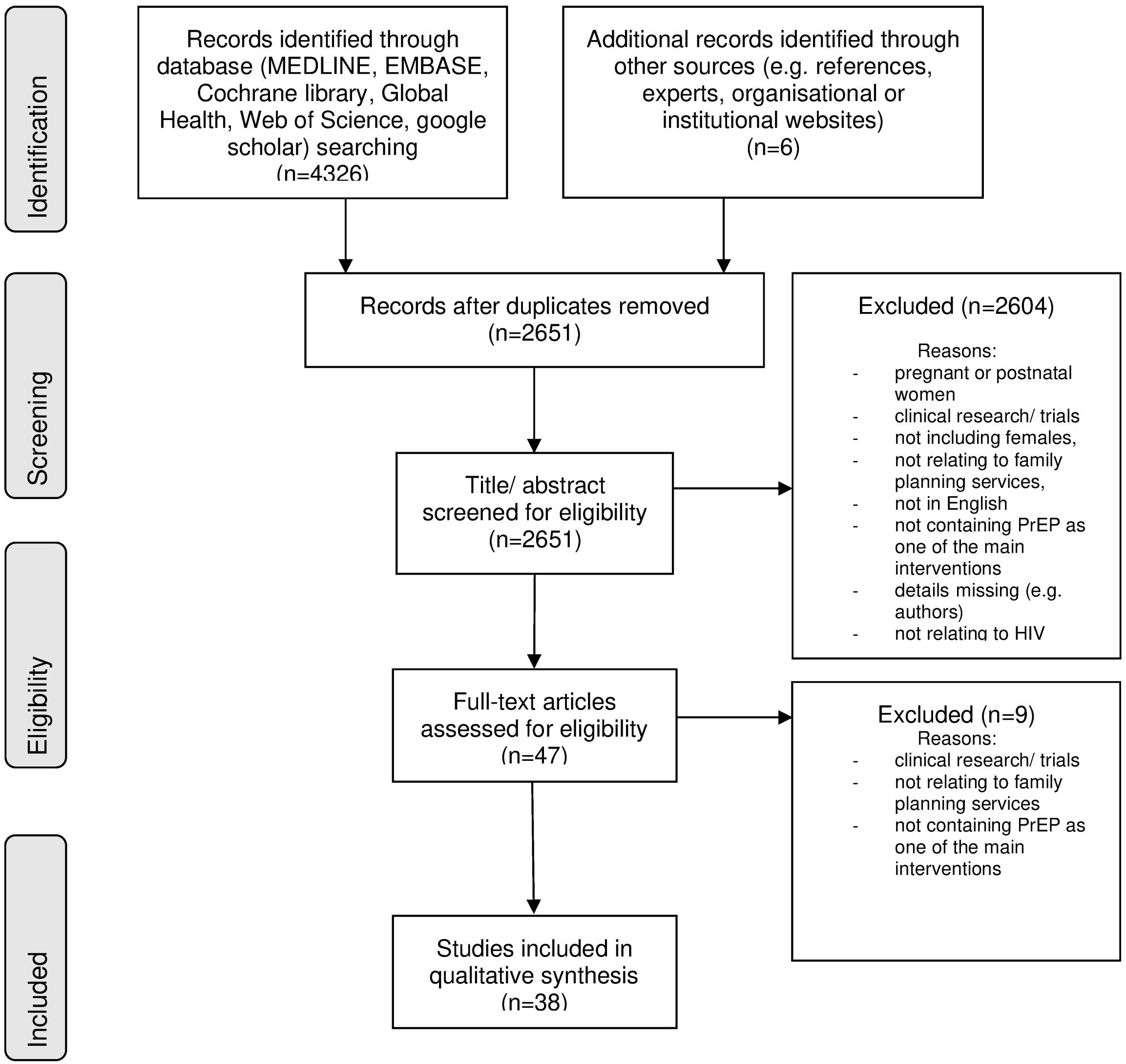

Supplement: Supplementary data [file bmjsrh-2021-201356supp001.pdf]
